# Frequency-Diverse Bunching Metamaterial Antenna for Coincidence Imaging

**DOI:** 10.3390/ma12111817

**Published:** 2019-06-04

**Authors:** Mengran Zhao, Shitao Zhu, Jianxing Li, Hongyu Shi, Juan Chen, Yuchen He, Anxue Zhang

**Affiliations:** School of Electronic and Information Engineering, Xi’an Jiaotong University, Xi’an 710049, China; zmr1993@stu.xjtu.edu.cn (M.Z.); jianxingli.china@xjtu.edu.cn (J.L.); honyo.shi1987@gmail.com (H.S.); yuchenhe@xjtu.edu.cn (Y.H.); anxuezhang@xjtu.edu.cn (A.Z.)

**Keywords:** frequency-diverse, metamaterial, bunching, coincidence imaging

## Abstract

A frequency-diverse bunching metamaterial antenna for coincidence imaging in the Ka band is proposed in this paper. The bunching metamaterial antenna includes a broadband circular array and a frequency-diverse bunching metalens. Firstly, in order to enhance the bunching characteristic, the broadband circular array is designed based on the 60-degree beamwidth design to generate radiation patterns from 32 GHz to 36 GHz. Then, types of metamaterial elements with different transmission phases are selected to form the frequency-diverse bunching metalens based on a random distribution design and gradient zoom coefficient design. Moreover, the bunching metamaterial antenna is constituted by loading the frequency-diverse bunching metalens to the broadband circular array, which can generate frequency-diverse bunching random radiation patterns with beamwidth less than 100 degrees from 32 GHz to 36 GHz. Furthermore, the performances of the bunching metamaterial antenna, including the reflection coefficient, the radiation efficiency, and the correlation coefficients of radiation patterns at different frequencies are evaluated. Finally, the coincidence imaging experiment is implemented using the bunching metamaterial antenna and the image of the target is reconstructed successfully. The design is verified by simulations and measurements.

## 1. Introduction

Nowadays, coincidence imaging in microwave frequencies is becoming more and more popular. But the difficulty of balancing the imaging efficiency, cost, and volume of the system limits the application of coincidence imaging in many situations. However, the adoption of a metamaterial aperture antenna provides a new method to solve this problem. Metamaterials are artificial structures that can be designed to exhibit specific electromagnetic properties not commonly found in nature [1,2]. A group from the University of California, San Diego (UCSD), experimentally verified the existence of left-handed materials using copper split-ring resonators (SRRs) and thin copper wires [3]. With the rapid development of metamaterial theory, the application of metamaterials such as metamaterial lenses [4,5,6], metamaterial electromagnetic cloaks [7], perfect metamaterial absorbers [8], and so on becomes more and more popular. What’s more, the combination of metamaterial and antenna has improved the performance of antennas such as low-profile high-gain patch antennas [9,10], compact microstrip antennas [11,12], dual-frequency and broadband circular patch antennas [13,14], and so on. The frequency-diverse metamaterial antenna is also a new kind of antenna that can generate radiation patterns with low correlation coefficients at different frequencies, which can be used as different measurement modes in coincidence imaging.

In 2013, Hunt and colleagues first proposed a metaimager that is capable of microwave imaging by leveraging metamaterial. They also fabricated a prototype to illustrate the feasibility of holographic imaging at microwave frequencies [15,16,17,18]. In 2015, Sleasman and colleagues presented a dynamic metamaterial aperture designed for microwave computational imaging schemes by leveraging a metamaterial element with two diodes connected to an external control circuit [19,20,21,22,23,24,25]. The dynamic metamaterial provided a new method to design a metamaterial aperture antenna. In 2016, Fromenteze an colleagues presented a 3D computational imaging system based on a mode-mixing cavity metamaterial aperture at microwave frequencies [26,27,28,29,30]. The mode-mixing cavity used a Sinai billiard to catalyze mode mixing, which brought forward a new method to design the feed system of the metamaterial aperture antenna. In 2016, Wu et al. proposed a method to enhance the performance of metamaterial aperture imaging through rotating the metamaterial aperture panel around the panel axis [31], which provided a new method to improve the measurement modes besides increasing the feed probes and using dynamic metamaterials. However, the radiation patterns of the metamaterial antennas mentioned above usually cover theta from $-90^{\circ }$ to $90^{\circ }$. The energy out of the region of interest (ROI) decreases the signal-to-noise ratio (SNR) of the ROI, which would reduce the imaging quality. In order to solve this problem, in 2018 we proposed a frequency-diverse transmission metamaterial aperture that can generate bunching random beams to increase the imaging distance [32].

However, the profile of the metamaterial aperture proposed in [32] is too high. In this paper, a new method that uses a 60-degree beamwidth design, random distribution design, and gradient zoom coefficient design is proposed in order to design the frequency-diverse bunching metamaterial antenna (BMA) with low profile. The BMA can generate frequency-diverse radiation patterns with a beamwidth less than 100 degrees from 32 GHz to 36 GHz. In Section 2, the broadband circular array (BCA) and the frequency-diverse bunching metalens (FBDM) are designed. Moreover, the BMA is constituted through loading the FBDM to the BCA. In Section 3, the performances of the BMA, including the reflection coefficient, the radiation efficiency, and the correlation coefficients of radiation patterns at different frequencies are evaluated. The design is verified by both simulations and measurements. In Section 4, an experiment of coincidence imaging using the BMA is implemented, and the image of target is reconstructed successfully.

## 2. Design of the BMA

In this section, the BCA and the FDBM are respectively designed. Then the FDBM is loaded to the BCA to constitute the BMA.

### 2.1. Design of the BCA

In order to acquire bunching radiation patterns from 32 GHz to 36 GHz, the BCA used as the excitation source should be broadband and the beamwidth should be narrow. However, the FDBM cannot be fully stimulated if the beamwidth is too narrow. Thus, the beamwidth of the BCA is optimized to 60 degrees (i.e., the 60-degree beamwidth design) through simulations, which maintains the bunching radiation pattern and the full stimulation of the FDBM simultaneously.

**Figure 1:**
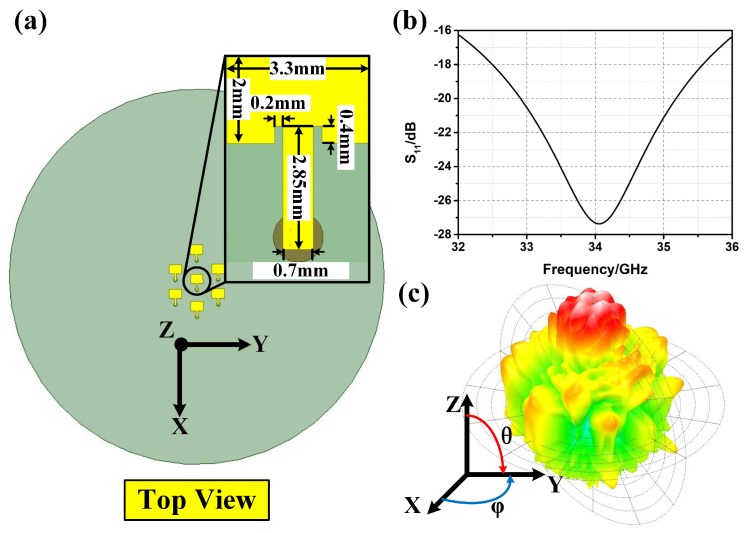
(**a**) The schematic of the broadband circular array (BCA); (**b**) the simulated reflection coefficient $S_{11}$ of the broadband antenna element; (**c**) the radiation pattern of the BCA.

As shown in Figure 1a, a patch antenna element fed by the coaxial probe is designed to meet the broadband requirement. The permittivity of the substrate is 2.55 and the thickness of the substrate is 1.58 mm. From the $S_{11}$ shown in Figure 1b, the patch antenna element is well fed from 32 GHz to 36 GHz. The BCA is composed of seven patch antenna elements (i.e., one inner antenna element and six outer antenna elements) as shown in Figure 1a. The position of the patch antenna is designed according to the theory of circular array and optimized using the High Frequency Structure Simulator (HFSS) software. The HFSS is a commercial software that can calculate electromagnetic fields utilizing the finite element method (FEM). The radius of the circular array is 7 mm. The radiation pattern of the designed circular array is shown in Figure 1c with a 60-degree beamwidth.

### 2.2. Design of the FDBM

According to the requirements of coincidence imaging, the radiation patterns of the BMA should be frequency-diverse (i.e., different frequencies corresponds to different radiation patterns).

**Figure 2:**
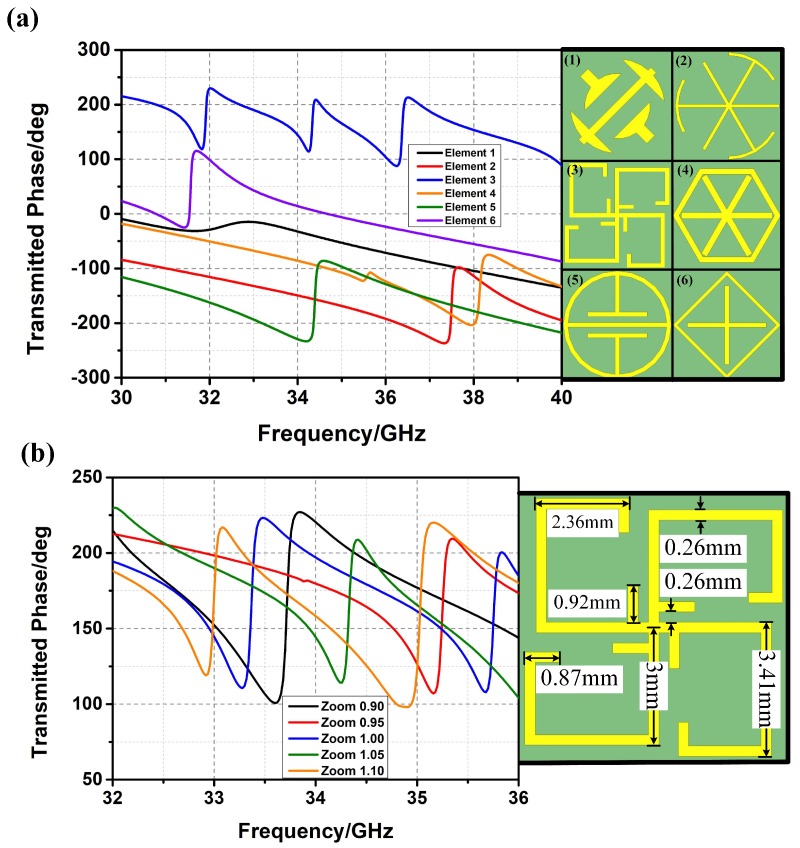
(**a**) Metamaterial elements with different transmission phases; (**b**) fractal metamaterial element with different zoom coefficients.

In order to achieve the frequency-diverse characteristic, metamaterial elements with different transmission phases from 32 GHz to 36 GHz, as shown in Figure 2a, are selected. From Figure 2a, different kinds of metamaterial elements have different transmission phases at the same frequency. Thus, different metamaterial elements distributed at different places would append different transmission phases when stimulated by the BCA, which results in frequency-diverse radiation patterns (i.e., a random distribution design).

In order to achieve the bunching characteristic of the FDBM, a quasi-gradient phase distribution of the metalens is required. Nevertheless, it is difficult to realize gradient phase and random phase simultaneously at the same frequency through designing the structure of metamaterial elements. However, by zooming out the metamaterial element, the turning point of the transmission phase can be moved to a high frequency, which inspired a new approach to achieve the bunching characteristic of the FDBM.

**Figure 3:**
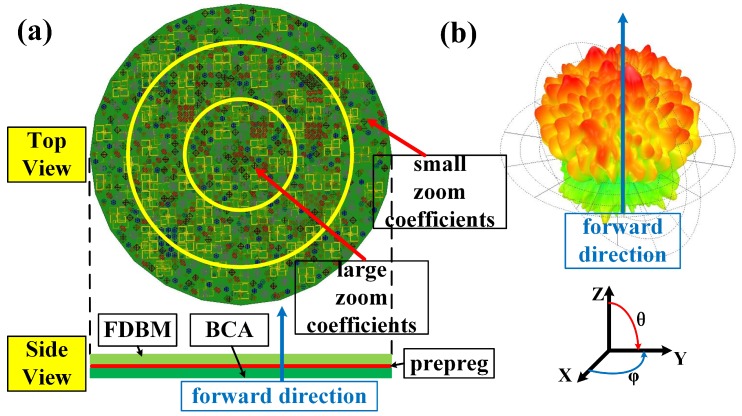
(**a**) The schematic of the bunching metamaterial antenna (BMA); (**b**) radiation pattern of the BMA at 34 GHz. FDBM, frequency-diverse bunching metalens.

The fractal metamaterial element is taken to illustrate the design of FDBM. The permittivity of the substrate is 2.65 and the thickness of the substrate is 1.58 mm. As shown in Figure 2b, transmission phases of fractal metamaterial elements with small zoom coefficients are above those with large zoom coefficients at most frequencies. Thus, the quasi-gradient phase is obtained through dividing regions with gradient zoom coefficients on the FDBM (i.e., a gradient zoom coefficient design) as shown in Figure 3a. The zoom coefficients of inner metamaterial elements are larger than those of outer metamaterial elements.

### 2.3. Design of the BMA

The BMA is composed of the BCA and the FDBM. The radiation pattern of the BMA at 34 GHz, shown in Figure 3b, is a bunching random radiation pattern. The radiation patterns of the BMA still maintain the bunching characteristic due to the 60-degree beamwidth design of the BCA and the gradient zoom coefficient design of the FDBM, while the frequency-diverse random beams are the result of the random distribution design of the FDBM.

**Figure 4:**
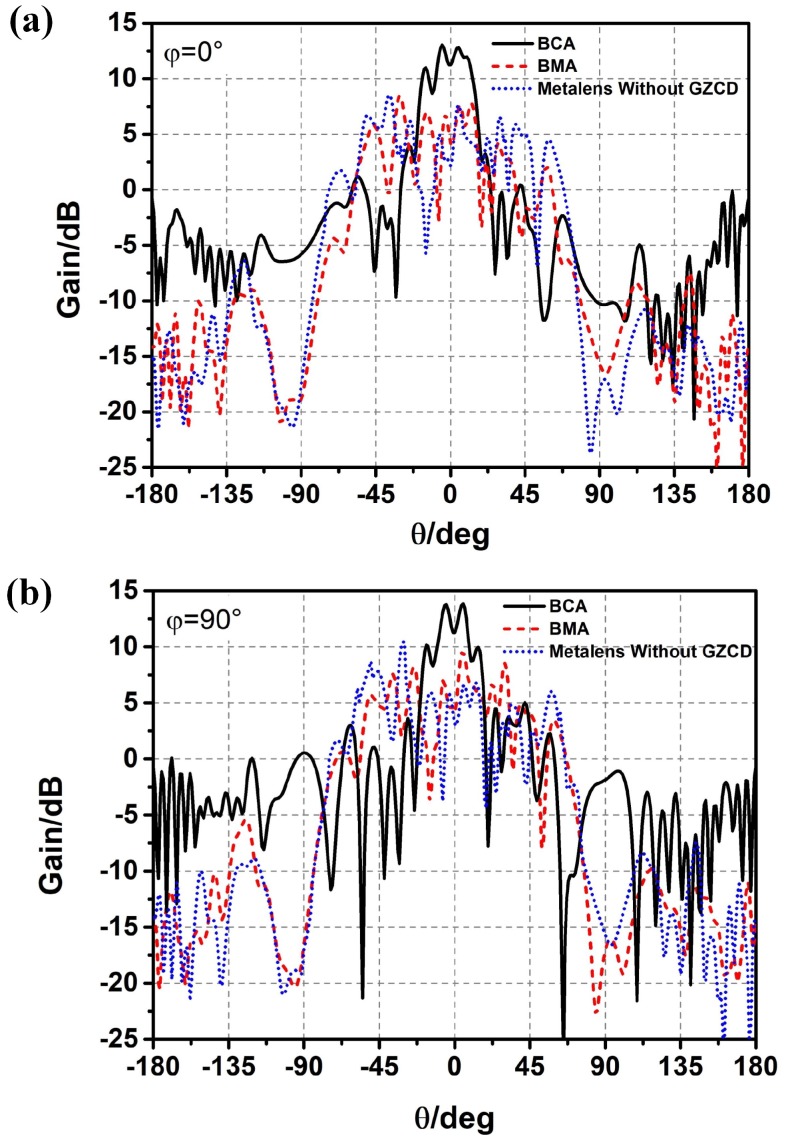
Comparison of radiation patterns of the BCA covered by a metalens without gradient zoom coefficient design (GZCD), the BCA, and the BMA; (**a**) $\varphi =0^{\circ }$; (**b**) $\varphi =90^{\circ }$.

The comparisons of radiation patterns of the BCA, the BMA, and the BCA covered by a metalens without a gradient zoom coefficient design are shown in Figure 4a,b. The conclusion can be drawn that the radiation pattern of the metamaterial aperture antenna can be bunched through the 60-degree beamwidth design and the gradient zoom coefficient design, which provides a new method for designing frequency-diverse bunching random radiation patterns.

## 3. Simulated and Measured Results

**Figure 5:**
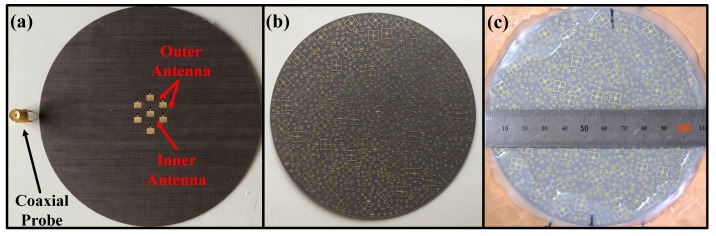
(**a**) The fabricated BCA; (**b**) the fabricated FDBM; (**c**) the fabricated BMA.

To validate the BMA, a prototype was fabricated as shown in Figure 5. The substrate of the BCA is TLX-8 of TACONIC with a permittivity of 2.55 and the substrate of the FDBM is TLX-6 of TACONIC with a permittivity of 2.65. The BCA is fed by seven coaxial probes as shown in Figure 5a. The FDBM (shown in Figure 5b) is stuck to the top of the BCA by hot-melt adhesive to constitute the BMA, as shown in Figure 5c. The diameter of the BMA is 110 mm, as shown in Figure 5c.

**Figure 6:**
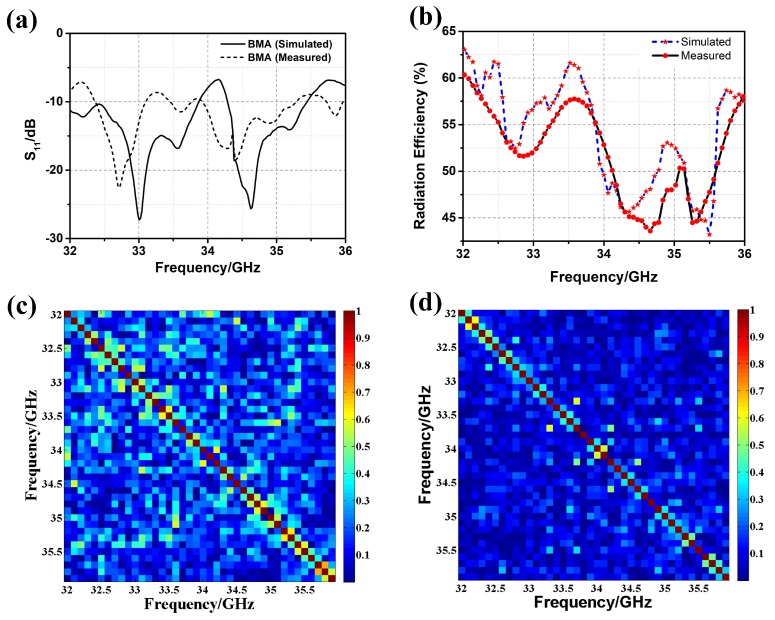
(**a**) $S_{11}$ of the BMA with/without metalens, both simulated and measured; (**b**) radiation efficiency of the BMA, both simulated and measured; (**c**) correlation coefficients of the simulated frequency-diverse radiation patterns; (**d**) orcrelation coefficients of the measured frequency-diverse radiation patterns.

The comparison of the simulated and measured $S_{11}$ (i.e., reflection coefficient) of the BMA is shown in Figure 6a. The $S_{11}$ was measured by the Agilent vector network analyzer (VNA) E8363B. The VNA and the coaxial cables were calibrated using short-open-load-thru (SOLT) calibration. The calibration kit was Agilent 85052D. Both the simulated and measured $S_{11}$ were under −10 dB from 32 GHz to 36 GHz, which proves that the BMA is well fed. The comparison of simulated and measured radiation efficiency is shown in Figure 6b. The average measured radiation efficiency was over 0.5 from 32 GHz to 36 GHz, which is a little lower than that of the simulated radiation efficiency. The BMA was designed for coincidence imaging that requires amounts of measurement modes. Different measurement modes used in coincidence imaging should be low-correlated [33,34]. Thus, the correlation coefficients of radiation patterns at different frequencies should be calculated as follows. Firstly, the amplitude and the phase of the radiation pattern at a specific frequency are arranged into two-dimensional matrices corresponding to the spatial distribution. Then, the amplitude matrix and the phase matrix constitute the complex matrix of the radiation pattern at this frequency. Finally, complex matrices of different frequencies are used to calculate the cross-correlations (i.e., the correlation coefficients of radiation patterns). The pixels, as shown in Figure 6c,d, represent the normalized correlation coefficients of the radiation patterns at the frequencies shown on the x-axis and the y-axis. As shown in Figure 6c, most correlation coefficients of the simulated frequency-diverse radiation patterns were under 0.4. Because of errors stemming from fabrication and measurement, most of the correlation coefficients of the measured frequency-diverse radiation patterns are under 0.3, as shown in Figure 6d, which is lower than those of the simulated radiation patterns. Both simulated and measured results promise good performance in the implementation of coincidence imaging.

## 4. Coincidence Imaging Using the BMA

The measurement modes (i.e., complex radiation patterns of the BMA at different center frequencies) are required in coincidence imaging. In the ideal case, the measurement modes are independent from each other. Most correlation coefficients of the measurement modes generated by the BMA are under 0.3 according to the measured results. Hence, the measurement equation matrix can be formed using all of the measured data [35]:

(1) $$\left[\begin{array}{c} S_{t}\left(t_{1}\right)\\ S_{t}\left(t_{1}\right)\\ \vdots \\ S_{t}\left(t_{M}\right) \end{array}\right]=\left[\begin{array}{cccc} S_{R}\left(I_{1},t_{1}\right) & S_{R}\left(I_{2},t_{1}\right) & \cdots & S_{R}\left(I_{Q},t_{1}\right)\\ S_{R}\left(I_{1},t_{2}\right) & S_{R}\left(I_{2},t_{2}\right) & \cdots & S_{R}\left(I_{Q},t_{2}\right)\\ \vdots & \vdots & \; & \vdots \\ S_{R}\left(I_{Q},t_{M}\right) & S_{R}\left(I_{Q},t_{M}\right) & \cdots & S_{R}\left(I_{Q},t_{M}\right) \end{array}\right]\left[\begin{array}{c} \sigma (I_{1})\\ \sigma (I_{2})\\ \vdots \\ \sigma (I_{Q}) \end{array}\right]+\left[\begin{array}{c} n(t_{1})\\ n(t_{2})\\ \vdots \\ n(t_{M}) \end{array}\right],$$

where $S_{t}\left(t_{m}\right)$ is the measurement data, $S_{R}\left(I_{q},t_{m}\right)$ is the distribution of the measurement mode in the imaging plane, $I_{q}$ represents the discrete imaging point, $\sigma (I_{q})$ is the scatting coefficient at the $I_{q}th$ imaging point, $t_{m}$ represents the index of the tests, and $n(t_{m})$ is the measurement noise. Equation (1) can be solved using the fast iterative shrinkage threshold algorithm (FISTA) method [36] or the conjugate gradient least squares algorithm (CGLSA), Ref. [37] with high efficiency. Thus, the high-quality image can be reconstructed using all of the measured data of the BMA.

**Figure 7:**
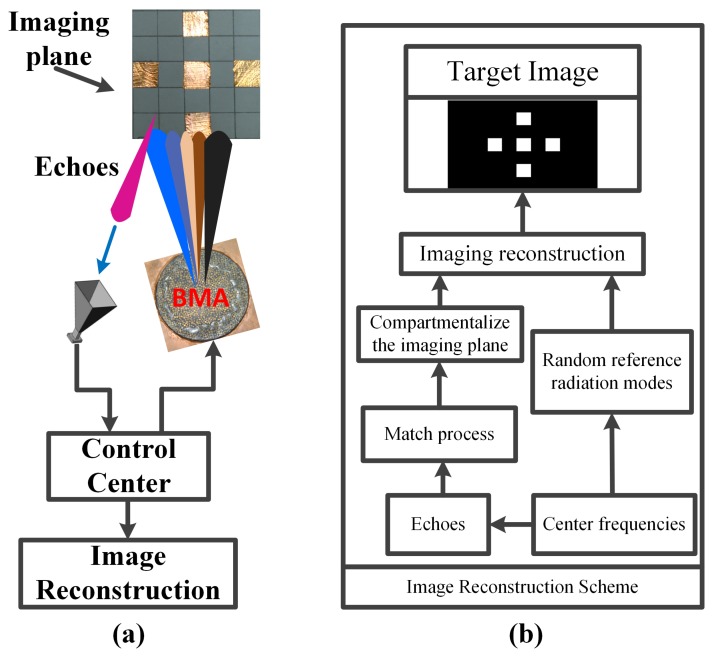
(**a**) Schematic of the coincidence imaging; (**b**) flowchart of the coincidence imaging.

The schematic of the coincidence imaging is shown in Figure 7a. The BMA is used as the excitation and a horn antenna is used as the receiver to obtain the echo data. The target is in the 3 dB beamwidth of bunching random beams generated by the BMA. The radius of the BMA is 55 mm. The imaging distance is 50 cm. The flowchart of the coincidence imaging is shown in Figure 7b. The match process includes the target distance estimation and the match between the measurement modes and the echoes.

**Figure 8:**
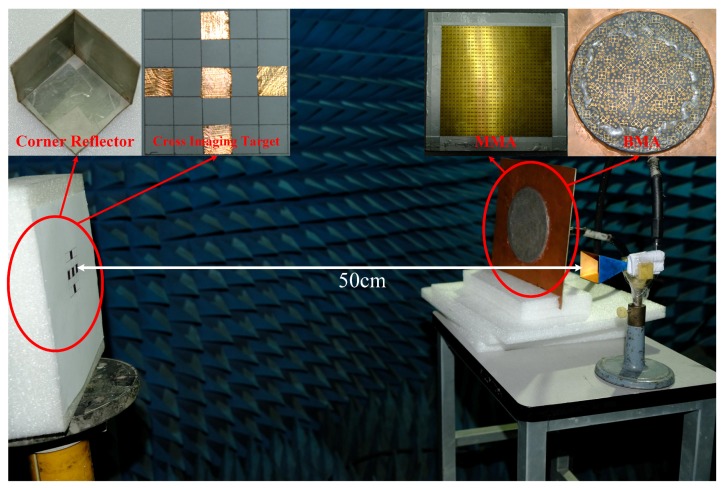
Coincidence imaging experiments. MMA, metamaterial aperture antenna.

**Figure 9:**
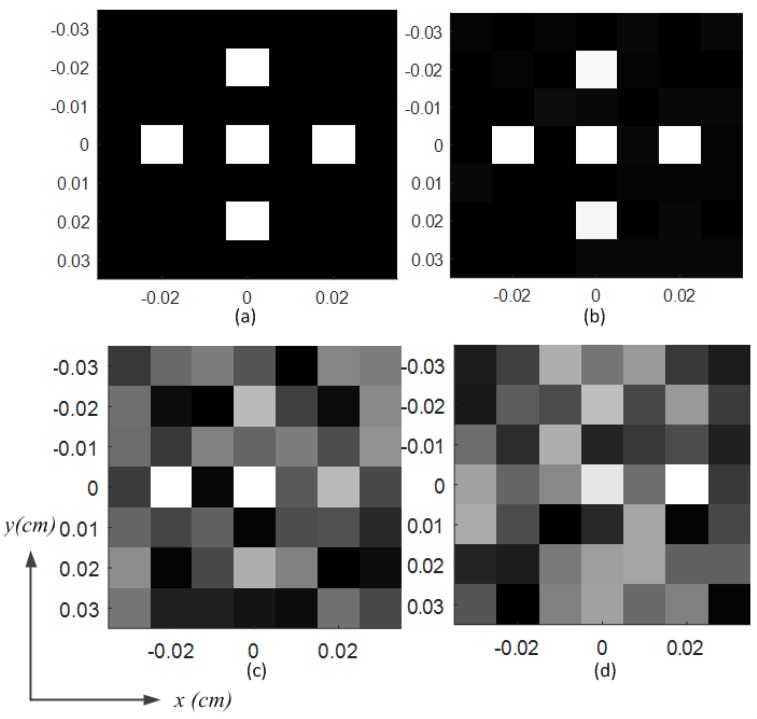
Comparisons of the imaging results; (**a**) origin image; (**b**) reconstructed image with a signal-to-noise ratio (SNR) of 20 dB; (**c**) reconstructed image with an SNR of 14 dB; (**d**) reconstructed image with an SNR of 8 dB.

The scene of the coincidence imaging experiment is shown in Figure 8. In order to show the advantages of the BMA, a metamaterial aperture antenna (MAA) without bunching characteristic proposed in [38] is selected for comparison. The side length of the MAA is 120 mm, which is approximately the diameter of the BMA. As shown in Figure 7, a corner reflector is used as the target to demonstrate the advantage of the bunching characteristic. The SNR of the receiving signal of the BMA is 6.3 dB higher than that of the MAA, which illustrates that the bunching characteristic can improve the SNR. Then the cross imaging target is placed at the imaging plane as shown in Figure 8. The imaging plane contains $K_{1}\times K_{2}$ imaging cells and is equally discretized, where $K_{1}$ is in the azimuth direction and $K_{2}$ is in the pitch direction. The side length of the imaging cells is 1 cm (about 1/5 of the 3 dB beamwidth of the coherent transmitting aperture with the same size as the BMA) in both directions. The quality of the image can be evaluated by the entropy. The entropy of an image is defined as [39]

(2) $$E=\sum_{x=1}^{X}\sum_{y=1}^{Y}D\left(x,y\right)\ln\left[D\left(x,y\right)\right],$$

where $D\left(x,y\right)=\left|d\left(x,y\right)\right|/\sum_{x=1}^{X}\sum_{y=1}^{Y}\left|d\left(x,y\right)\right|$, d(x,y) is the image data with coordinates (x,y) in the imaging plane, and X×Y is the image area. As shown in Figure 9, the image of the target is reconstructed by BMA. The origin image is shown in Figure 9a. The image of the target can be reconstructed with high quality when the SNR is bigger than 20 dB, as shown in Figure 9b. The quality of the reconstructed image declines with the decreases of the SNR. As shown in Figure 9c, the reconstructed image of the target can be distinguished when the SNR is 14 dB. When the SNR is lower than 8 dB, the reconstructed image is completely blurred, as shown in Figure 9d. Through twenty independent coincidence imaging experiments, the average entropies of the reconstructed image under the same conditions as Figure 9b–d are 2.32, 3.51, and 3.74, respectively.

**Figure 10:**
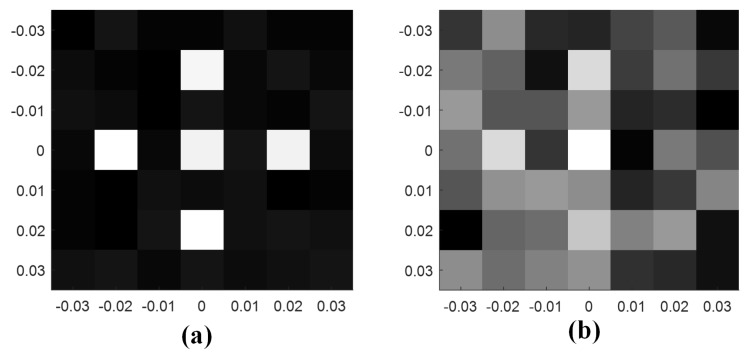
Comparison of the imaging results under the same experiment conditions; (**a**) Reconstructed image using the BMA; (**b**) Reconstructed image using the MMA.

Figure 10 shows the comparison of the reconstructed images using the BMA and metamaterial aperture antenna (MMA) under the same experiment condition. Through twenty independent coincidence imaging experiments, the average entropies of the reconstructed image as shown in Figure 10a,b are 2.88 and 3.68, respectively. The comparison coincidence imaging experiment illustrates the advantages of BMA.

## 5. Conclusions

In this paper, a frequency-diverse bunching metamaterial antenna (BMA) for coincidence imaging in the Ka band has been proposed. The BMA was composed of the broadband circular array (BCA) and the frequency-diverse bunching metalens (FDBM). Firstly, the broadband antenna element fed by the coaxial probe was designed to achieve a good match from 32 GHz to 36 GHz. Furthermore, the circular array was designed based on the 60-degree beamwidth design to enhance the bunching characteristic. Then, based on a random distribution design and gradient zoom coefficient design, the FDBM was composed of several metamaterial elements with different transmission phases. Moreover, the FDBM was loaded to the BCA to constitute the BMA. Finally, a coincidence imaging experiment using the BMA was implemented and the image of the target was reconstructed. The performances of the BMA were validated through simulations and measurements.

## Abbreviations

The following abbreviations are used in this manuscript:

BMA	Bunching metamaterial antenna
BCA	Broadband circular array
FDBM	Frequency-diverse bunching metalens
HFSS	High Frequency Structure Simulator
FEM	Finite element method
ROI	Region of interest
SNR	Signal-to-noise ratio
GZCD	Gradient zoom coefficient design
VNA	Vector network analyzer
SOLT	Short-open-load-thru
FISTA	Fast iterative shrinkage threshold algorithm
CGLSA	Conjugate gradient least squares algorithm
MMA	Metamaterial aperture antenna
